# Supplementary material for: Animal Age Affects the Gut Microbiota and Immune System in Captive Koalas (Phascolarctos cinereus)
Source: Microbiol Spectr. 2023 Jan 5;11(1):e04101-22. doi: 10.1128/spectrum.04101-22 (PMC9927321; doi:10.1128/spectrum.04101-22)
Supplement: Supplemental file 1 — Fig. S1 to S7. Download spectrum.04101-22-s0001.pdf, PDF file, 1.4 MB [file spectrum.04101-22-s0001.pdf]

Supplemental Materials

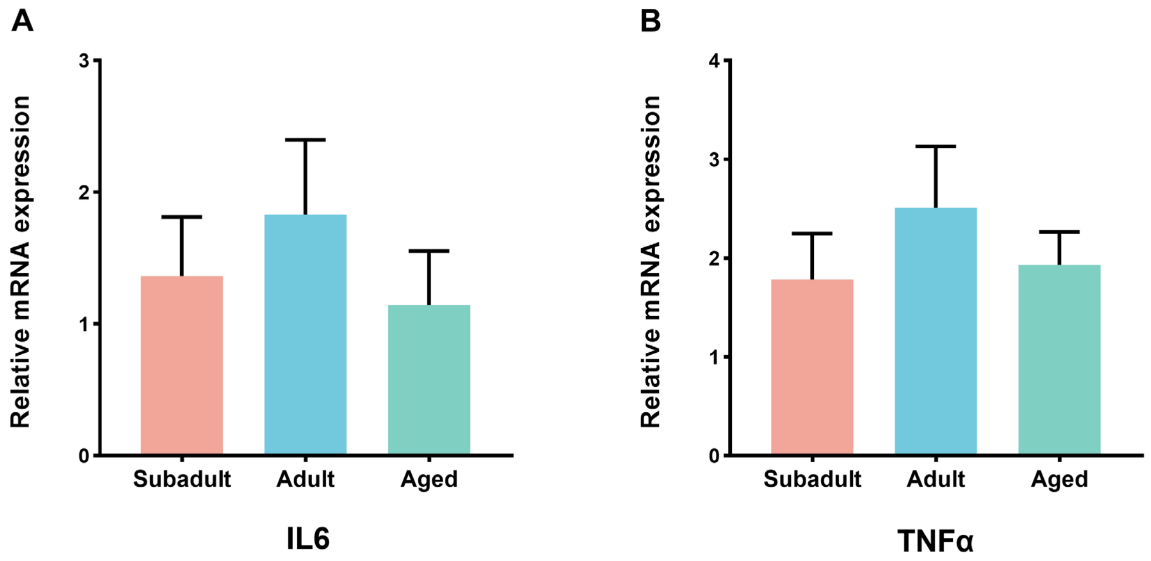

Supplemental Figure 1. Expression profiles of the blood immune genes in three age groups. (A) IL-6 and (B) TNFα. Mean values ± SEM are shown.

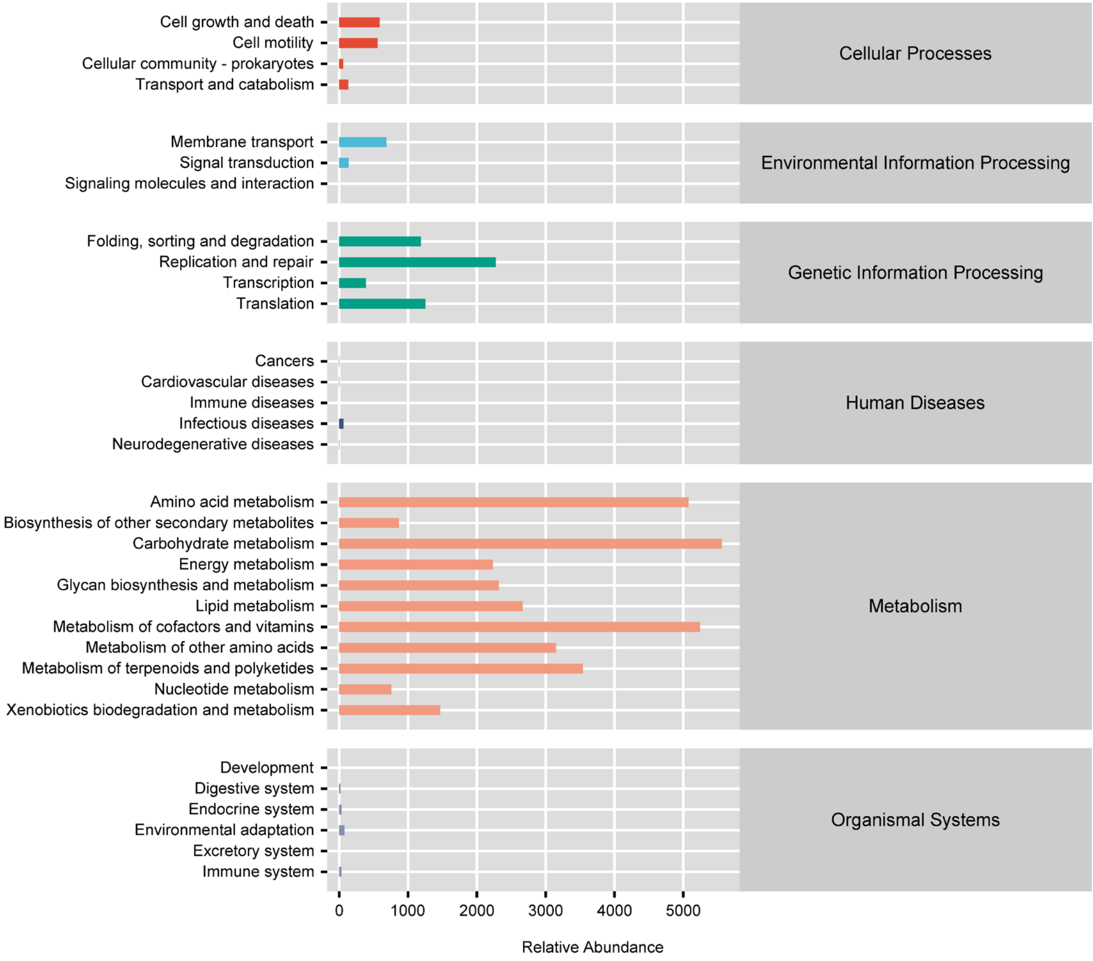

Supplemental Figure 2 Age-associated gut microbial functional predicted KEGG pathways.

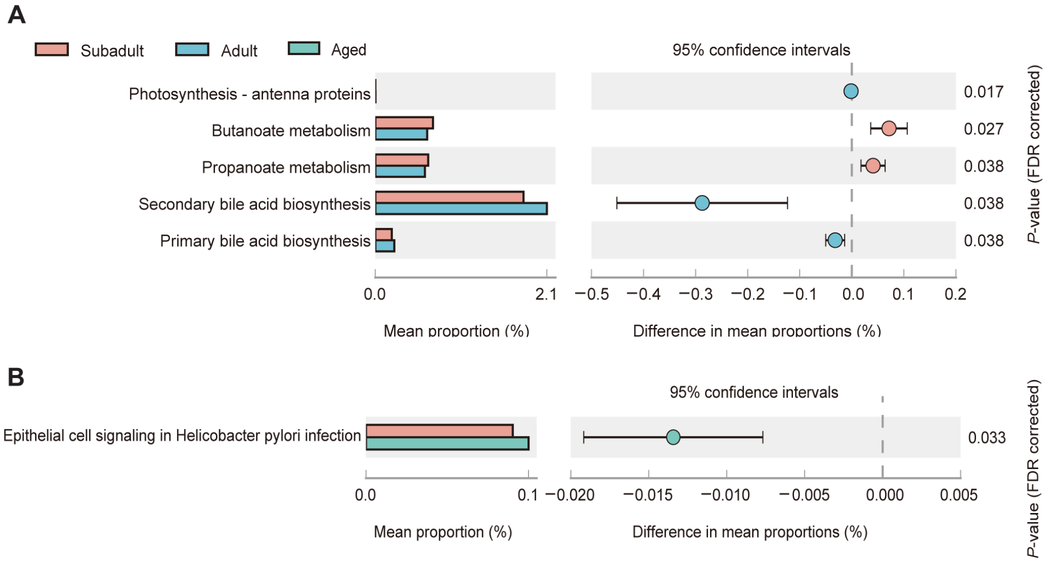
A
Subadult
Adult
Aged
95% confidence intervals
Photosynthesis - antenna proteins
Butanoate metabolism
Propanoate metabolism
Secondary bile acid biosynthesis
Primary bile acid biosynthesis
0.017
0.027
0.038
0.038
0.038
0.0
2.1
−0.5
−0.4
−0.3
−0.2
−0.1
0.0
0.1
0.2
Mean proportion (%)
Difference in mean proportions (%)
P-value (FDR corrected)
B
95% confidence intervals
Epithelial cell signaling in Helicobacter pylori infection
0.033
0.0
0.1
−0.020
−0.015
−0.010
−0.005
0.000
0.005
Mean proportion (%)
Difference in mean proportions (%)
P-value (FDR corrected)

10

C

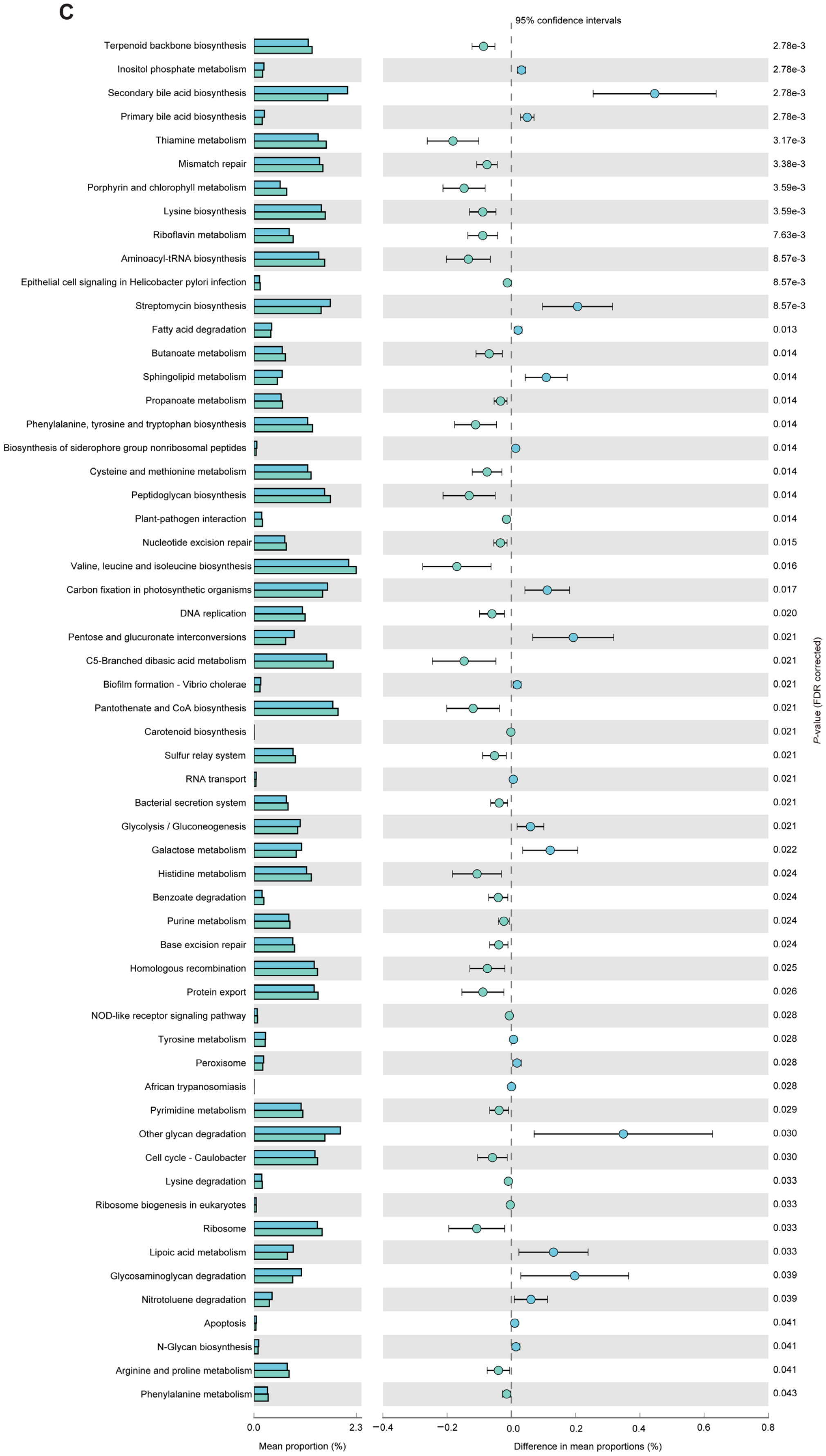
95% confidence intervals
Terpenoid backbone biosynthesis
Inositol phosphate metabolism
Secondary bile acid biosynthesis
Primary bile acid biosynthesis
Thiamine metabolism
Mismatch repair
Porphyrin and chlorophyll metabolism
Lysine biosynthesis
Riboflavin metabolism
Aminoacyl-tRNA biosynthesis
Epithelial cell signaling in Helicobacter pylori infection
Streptomycin biosynthesis
Fatty acid degradation
Butanoate metabolism
Sphingolipid metabolism
Propanoate metabolism
Phenylalanine, tyrosine and tryptophan biosynthesis
Biosynthesis of siderophore group nonribosomal peptides
Cysteine and methionine metabolism
Peptidoglycan biosynthesis
Plant-pathogen interaction
Nucleotide excision repair
Valine, leucine and isoleucine biosynthesis
Carbon fixation in photosynthetic organisms
DNA replication
Pentose and glucuronate interconversions
C5-Branched dibasic acid metabolism
Biofilm formation - Vibrio cholerae
Pantothenate and CoA biosynthesis
Carotenoid biosynthesis
Sulfur relay system
RNA transport
Bacterial secretion system
Glycolysis / Gluconeogenesis
Galactose metabolism
Histidine metabolism
Benzoate degradation
Purine metabolism
Base excision repair
Homologous recombination
Protein export
NOD-like receptor signaling pathway
Tyrosine metabolism
Peroxisome
African trypanosomiasis
Pyrimidine metabolism
Other glycan degradation
Cell cycle - Caulobacter
Lysine degradation
Ribosome biogenesis in eukaryotes
Ribosome
Lipoic acid metabolism
Glycosaminoglycan degradation
Nitrotoluene degradation
Apoptosis
N-Glycan biosynthesis
Arginine and proline metabolism
Phenylalanine metabolism
2.78e-3
2.78e-3
2.78e-3
2.78e-3
3.17e-3
3.38e-3
3.59e-3
3.59e-3
7.63e-3
8.57e-3
8.57e-3
8.57e-3
0.013
0.014
0.014
0.014
0.014
0.014
0.014
0.014
0.014
0.015
0.016
0.017
0.020
0.021
0.021
0.021
0.021
0.021
0.021
0.021
0.021
0.021
0.022
0.024
0.024
0.024
0.024
0.025
0.026
0.028
0.028
0.028
0.028
0.029
0.030
0.030
0.033
0.033
0.033
0.033
0.039
0.039
0.041
0.041
0.041
0.043
P-value (FDR corrected)
0.0
2.3
Mean proportion (%)
−0.4
−0.2
0.0
0.2
0.4
0.6
0.8
Difference in mean proportions (%)

Supplemental Figure 3. Bacterial functional pathways that are significantly associated with age at KO level 3. (A) Subadult compared to Adult, (B) Subadult compared to Aged, and (C) Adult compared to Aged. The bar plot shows the mean proportions of differential KEGG pathways predicted using PICRUSt2. The difference in proportions between the groups is shown with 95% confidence intervals. Only *P* value < 0.05 (Welch's t-test, FDR adjusted) are shown and composition.

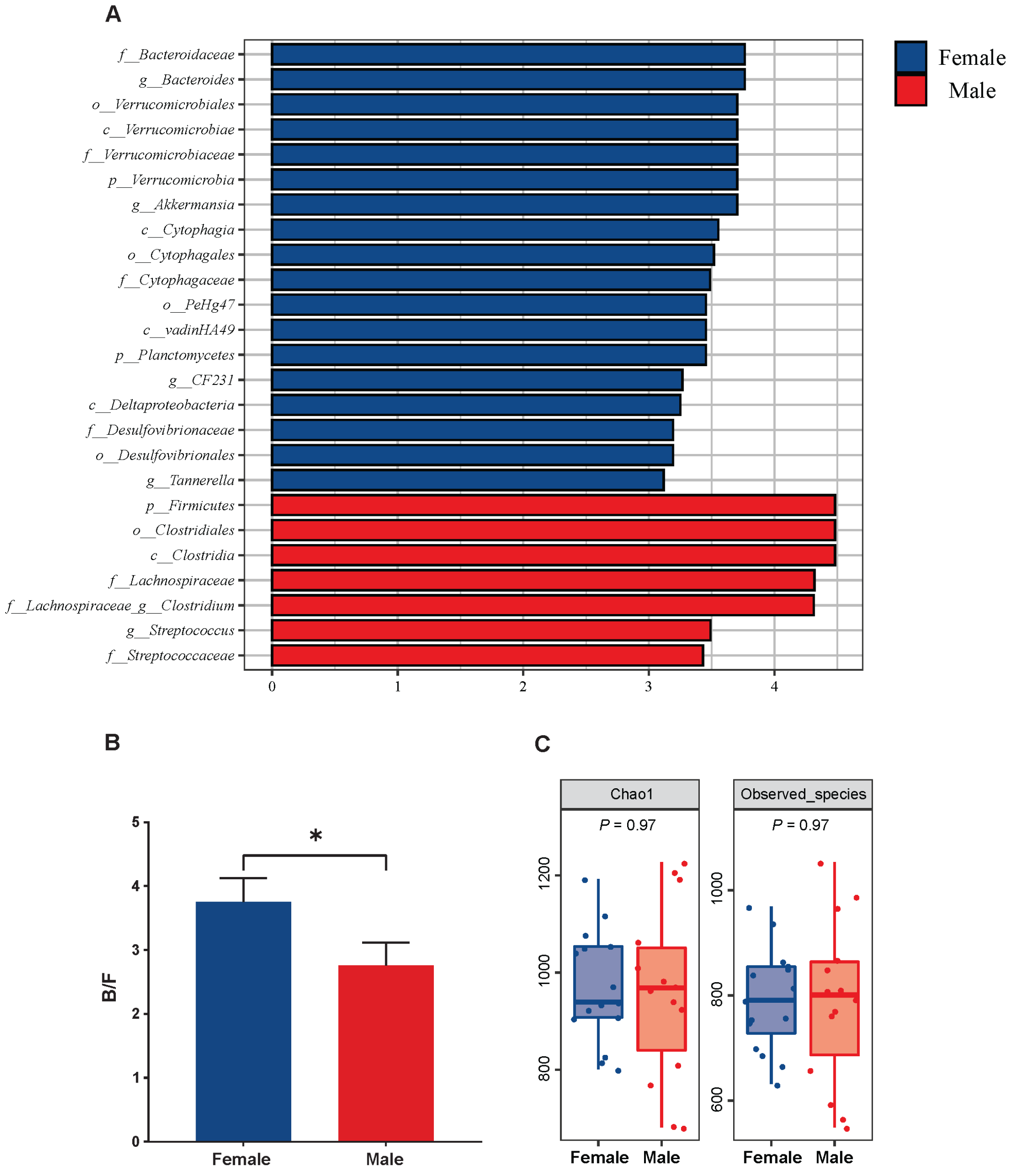

Supplemental Figure 4. Effect of sex on the koala's gut microbiota. (A) The bar chart shows differentially abundant taxa in the female and male ($P < 0.05$, LDA > 2). (B) The relative proportion of *Bacteroidetes* to *Firmicutes* (B/F) ratio. (C) Alpha diversity index (Chao1 index and Observed species diversity). Mean values ± SEM are shown. The significance of the difference between groups tested by nonparametric Mann–Whitney U test with Bonferroni post-hoc test. *$P < 0.05$.

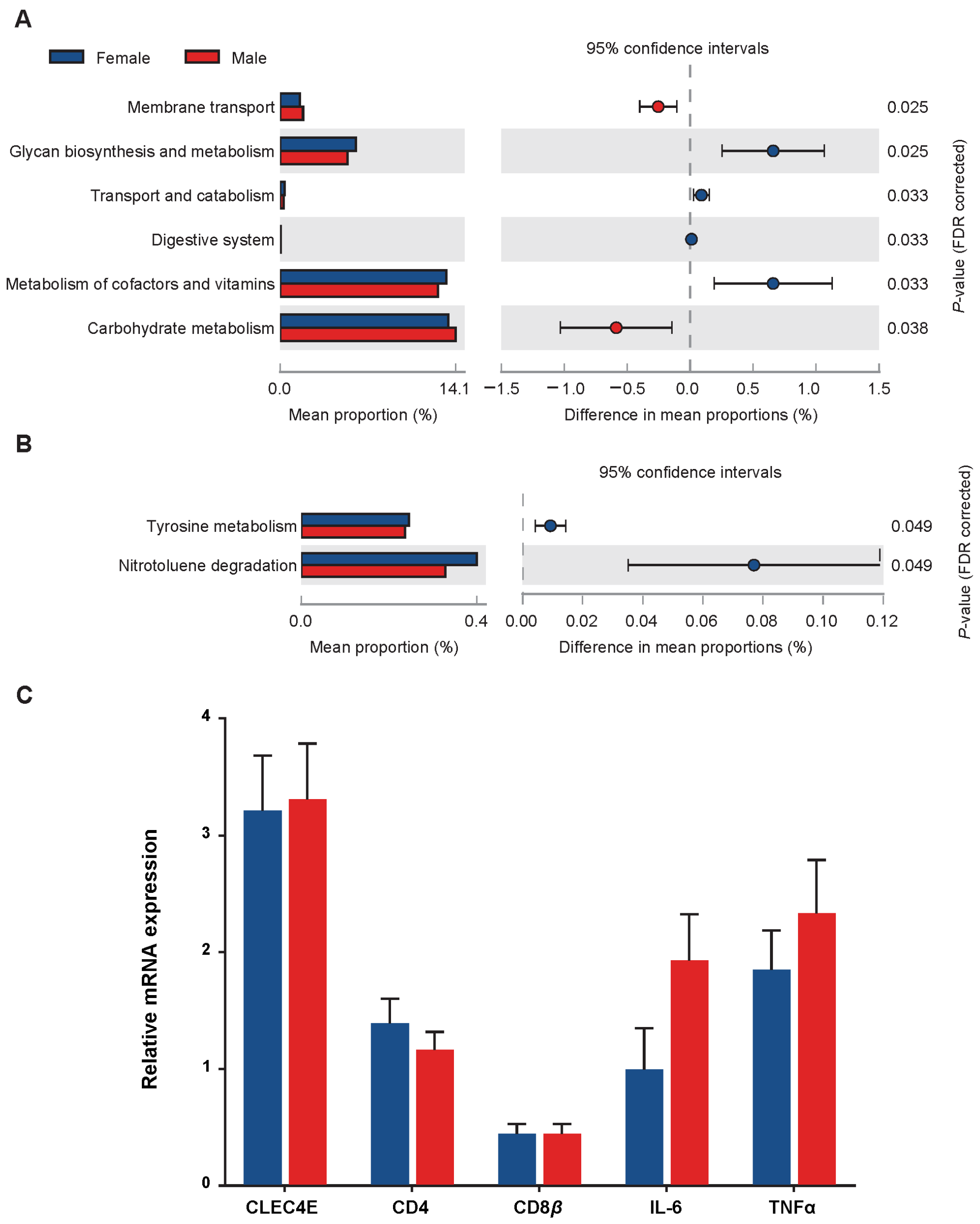

Supplemental Figure 5. Effect of sex on the koala's bacterial function and expression of the blood immune gene. (A) Bacterial pathways at level 2 of KEGG Orthology (KO) differ in proportions in female and male groups. (B) Bacterial pathways at level 3 of KO differ in proportions in female and male groups. The bar plot shows the mean proportions of differential KEGG pathways predicted using PICRUSt2. The difference in proportions between the groups is shown with 95% confidence intervals. Only P value < 0.05

(Welch's t-test, FDR adjusted) are shown and composition. (C) The expression of CLEC4E, CD4, CD8β, IL-6, and TNFα. Mean values ± SEM are shown.

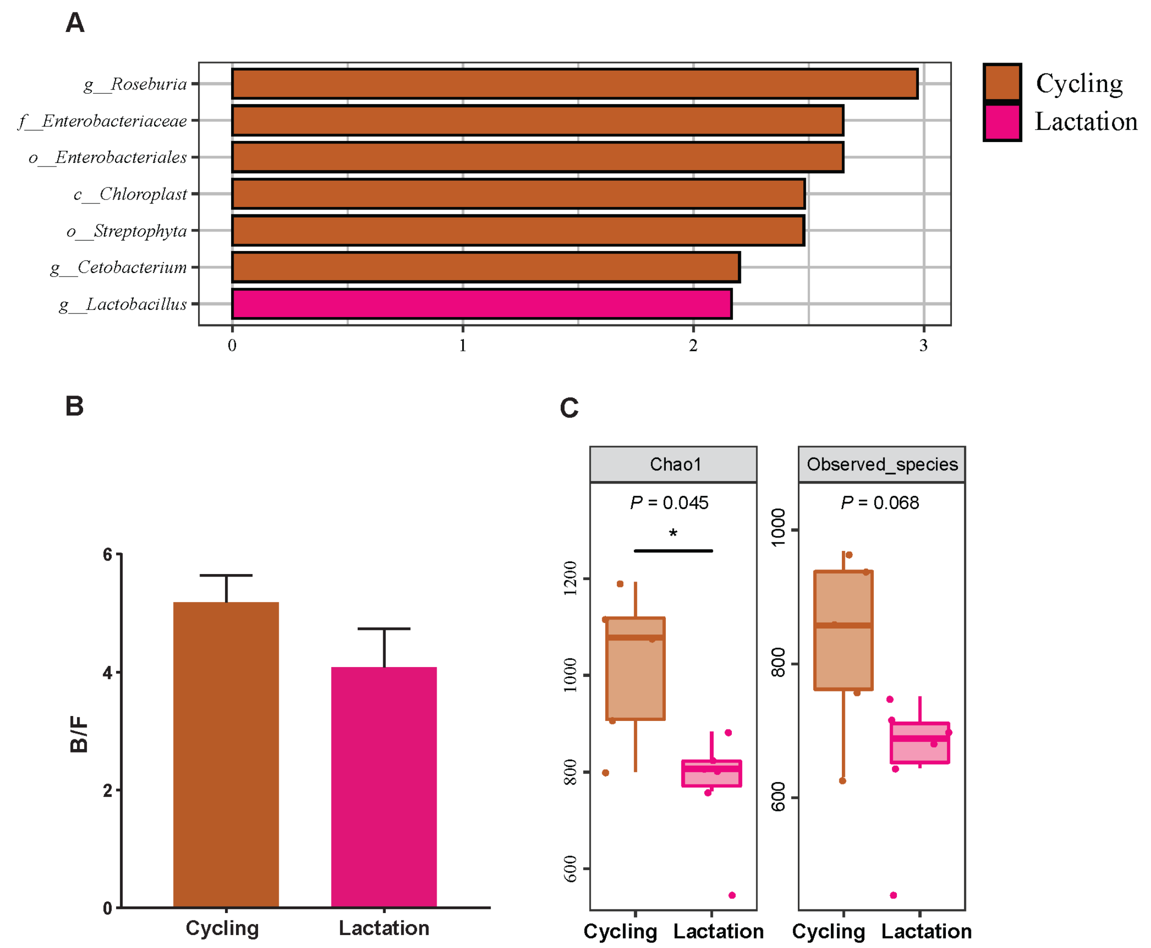

Supplemental Figure 6. Effect of female reproductive state on the koala's gut microbiota. (A) The bar chart shows differentially abundant taxa in the cycling female and lactation females ($P < 0.05$, LDA > 2). (B) The relative proportion of *Bacteroidetes* to *Firmicutes* (B/F) ratio. (C) Alpha diversity index (Chao1 index and Observed species diversity). Mean values ± SEM are shown. The significance of the difference between groups tested by nonparametric Mann–Whitney U test with Bonferroni post-hoc test. *$P < 0.05$.

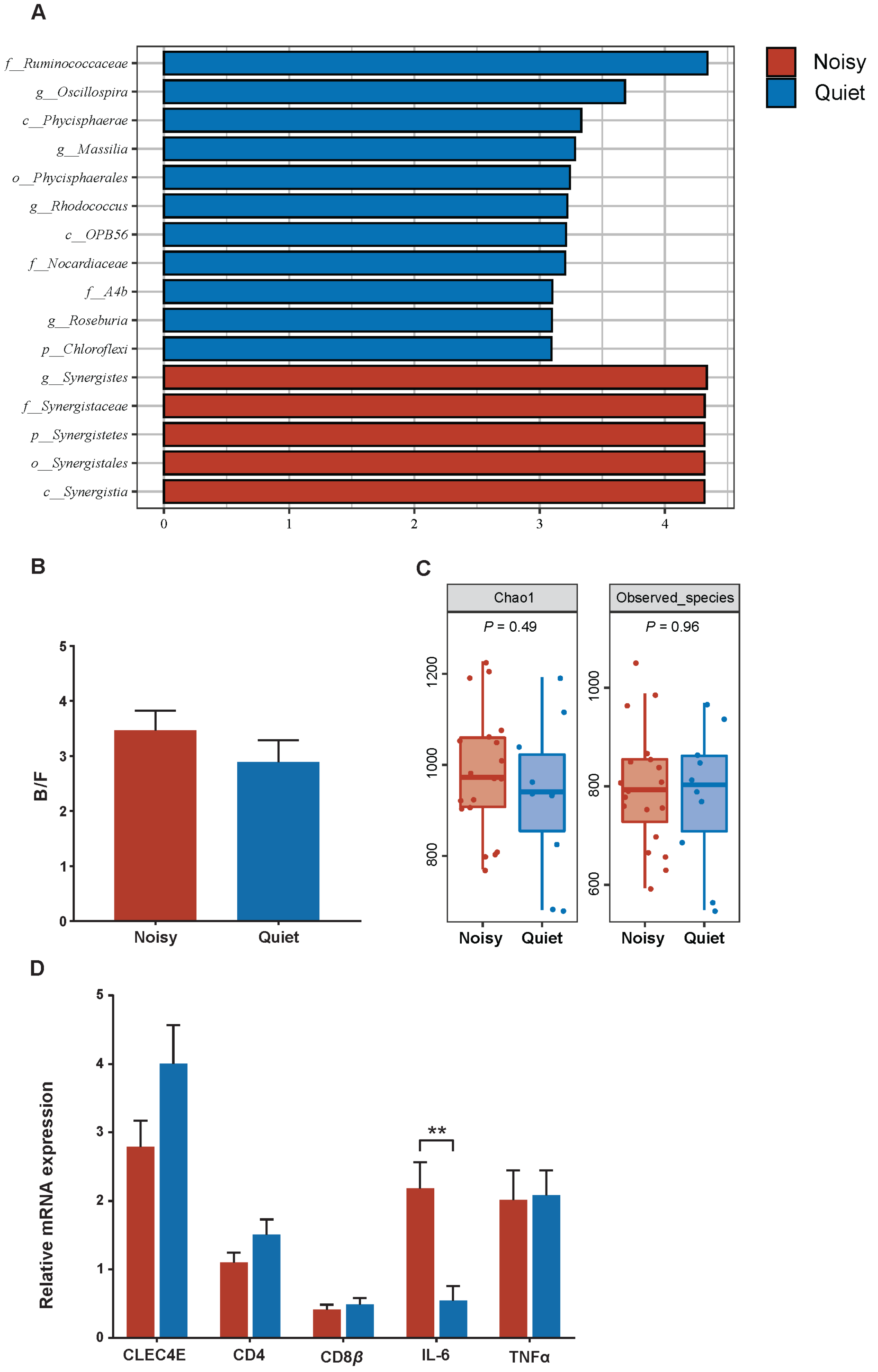
A
f__Ruminococcaceae
g__Oscillospira
c__Phycisphaerae
g__Massilia
o__Phycisphaerales
g__Rhodococcus
c__OPB56
f__Nocardiaceae
f__A4b
g__Roseburia
p__Chloroflexi
g__Synergistes
f__Synergistaceae
p__Synergistetes
o__Synergistales
c__Synergistia
0
1
2
3
4
Noisy
Quiet
B
B/F
Noisy
Quiet
C
Chao1
P = 0.49
Observed_species
P = 0.96
Noisy
Quiet
D
Relative mRNA expression
**
CLEC4E
CD4
CD8β
IL-6
TNFα

Supplemental Figure 7. Effect of living environment on the koala's gut microbiota and expression profiles of the blood immune genes. (A) The bar chart shows differentially abundant taxa in noisy (tourist regions) and quiet habitats ($P < 0.05$, LDA > 2). (B) The relative proportion of *Bacteroidetes* to *Firmicutes* (B/F) ratio. (C) Alpha diversity index (Chao1 index and Observed species diversity). (D) The expression of CLEC4E, CD4, CD8β, IL-6, and TNFα. Mean values ± SEM are shown. The significance of the difference between groups tested by ANOVA test with LSD post-hoc test. **$P < 0.01$.
